# Feasibility of Tomotherapy-Based Image-Guided Radiotherapy for Locally Advanced Oropharyngeal Cancer

**DOI:** 10.1371/journal.pone.0060268

**Published:** 2013-03-28

**Authors:** Nam P. Nguyen, Misty Ceizyk, Paul Vos, Michael Betz, Alexander Chi, Fabio Almeida, Rick Davis, Benjamin Slane, Steven Gelumbauskas, Lexie Smith-Raymond, Dave Abraham, Michelle Stevie, Siyoung Jang, Vincent Vinh-Hung

**Affiliations:** 1 Department of Radiation Oncology, University of Arizona, Tucson, Arizona, United States of America; 2 Department of Biostatistics, East Carolina University, Greenville, North Carolina, United States of America; 3 Hirslanden Radiation Oncology Institute, Lausanne, Switzerland; 4 Department of Radiation Oncology, West Virginia University, Morgantown, West Virginia, United States of America; 5 Southwest PET-CT Institute, Tucson, Arizona, United States of America; 6 Department of Radiation Oncology, University Hospitals of Geneva, Geneva, Switzerland; NIH, United States of America

## Abstract

**Purpose:**

The study aims to assess the feasibility of tomotherapy-based image-guided (IGRT) radiotherapy for locally advanced oropharyngeal cancer. A retrospective review of 33 patients undergoing concurrent chemoradiation for locally advanced oropharyngeal cancers was conducted. Radiotherapy planning, treatment toxicity and loco-regional control were assessed.

**Results:**

At a median follow-up of 32 months (6–47 months), no patient developed loco-regional recurrence. Two patients (6%) developed distant metastases. Grade 3–4 acute toxicity was respectively 72% and 25% for mucositis and gastrointestinal toxicity. Two patients (6%) had long-term dependence on tube feedings. Dose-volume histogram demonstrated excellent target volume coverage and low radiation dose to the organs at risk for complications.

**Conclusions and clinical relevance:**

IGRT provides excellent loco-regional control but acute toxicity remains significant and needs to be addressed in future prospective trials. The feasibility of Tomotherapy to decrease radiation dose to the normal tissues merits further investigations.

## Introduction

The prevalence of oropharyngeal cancer is steadily rising in the United States despite a reduction of other head and neck cancers [1]. The increase in oropharyngeal cancers is mainly observed in young patients and related to human papilloma virus (HPV) 16 which may reach epidemic proportion [2]. Most patients with oropharyngeal malignancyare frequently diagnosed at advanced stages because the submucosal spread of the tumor making it difficult to detect clinically despite patient's symptoms [3]. Locally advanced oropharyngeal cancers can be treated with either surgery followed by postoperative radiation or concurrent chemoradiation with similar survival [4]. Resection of the tongue base or soft palate is frequently associated with significant morbidity because of the crucial role of these organs in speech and deglutition [5], [6]. In addition, neck dissection, often bilateral, may induce severe pain because of nerve damage which may decrease patient quality of life [7]. Thus, concurrent chemoradiation is frequently selected over surgery for patients with locally advanced oropharyngeal cancers because of anatomic organ preservation [8]. However, chemoradiation is associated with significant toxicity, mainly grade 3–4 mucositis and hematologic toxicity, and long-term dysphagia because of damage to the pharyngeal muscles [9], [10]. New radiotherapy technique such as intensity-modulated radiotherapy (IMRT) has been introduced to decrease treatment toxicity in oropharyngeal cancers with promising preliminary results despite a short follow-up [11]. Image-guided radiotherapy (IGRT) is a new technique of IMRT delivery which combines the sharper dose fall off of IMRT and precise target irradiation [12]. The feasibility of IGRT has not been fully investigated in locally advanced oropharyngeal cancer and prompt us to conduct this retrospective study.

## Materials and Methods

The medical records of 33 patients undergoing radiotherapy for locally advanced oropharyngeal cancers at the University of Arizona Radiation Oncology department were retrospectively reviewed following institutional review board (IRB) approval from the University of Arizona. The University of Arizona IRB waived the requirement for patient consent because of the retrospective nature of the study limited to charts review. Patient information was de-identified to protect patient confidentiality. All patients were treated with the whole field IGRT technique on the helical Tomotherapy unit from December 2008 to February 2011. Prior to treatment, each patient was simulated in the supine position with a head and neck aquaplast mask for treatment immobilization. A computed tomography (CT) scan with and without intravenous (IV) contrast for treatment planning was performed in the treatment position. The head and neck areas from the vertex to the mid thorax were scanned with a slice thickness of 3 mm CT scan with IV contrast was employed to outline the tumor and grossly enlarged cervical lymph node for target volume delineation. Radiotherapy planning was performed on the CT scan without contrast to avoid possible interference of contrast density on radiotherapy isodose distributions. Diagnostic positron emission tomography (PET)-CT scan planning for tumor imaging was also incorporated with CT planning when available for tumor imaging. A 0.5 cm bolus material was placed on any area of the skin involved by the tumor and on any palpable cervical lymph nodes. Normal organs at risk for complication were outlined for treatment planning (spinal cord, brain stem, bilateral cochlea, mandible, parotid glands, bilateral eyes, and oral cavity).

For patients with definitive chemoradiation, the tumor and grossly enlarged lymph nodes (CTV1) on CT scan with a margin (PTV1) were treated to 70 Gy in 35 fractions (2 Gy/fraction). The margins were 5 mm to 1 cm all around CTV1 depending on anatomic location. The areas at high risk-PTV2 (at least 1 cm around gross tumor and pathologic cervical lymph nodes) and low risk -PTV3 (subclinical regional lymph nodes with 5 mm margins) for tumor spread were treated respectively to 63 Gy and 56 Gy in 35 fractions, respectively. Patients undergoing postoperative chemoradiation were treated to 66 Gy, 59.4 Gy, and 54 Gy in 33 fractions to PTV1, PTV2, and PTV3 respectively. Indications for postoperative chemoradiation were positive margins and/or extra-capsular lymph nodes invasion. Minimal target coverage was 95% of the prescribed dose for all targets with at least 99% of the prescribed dose delivered to gross tumor and involved cervical lymph nodes. The lymph nodes in the ipsilateral neck including the retropharyngeal lymph nodes were treated to the base of skull if there was any cervical lymph node enlargement (or PET-positive lymph nodes). Contralateral uninvolved lymph nodes were treated prophylactically with the C1 vertebrae as the superior border of the radiation field. In case of bilateral cervical lymph node involvement, both sides of the neck were irradiated including the base of skull to avoid any marginal miss. Mean dose to the parotid was kept below 2600 cGy if there was no ipsilateral cervical lymph node enlargement. Dose constraints for other normal organs at risk (OAR) for complications were: spinal cord (45 Gy), brain stem (50 Gy), optic chiasm (45 Gy), mandible (70 Gy to less than 30% of the mandible).

Concurrent chemoradiation was recommended for all patients. The type of chemotherapy regimen was left at the discretion of the medical oncologists depending on patient functional status and co-morbidities. Prophylactic percutaneous gastrostomy tubes feedings placement was recommended for all patients prior to radiotherapy because of the expected weight loss secondary to chemoradiation-induced mucositis. Weekly complete blood count (CBC) and blood chemistry to assess renal function were performed during chemoradiation. Treatment breaks and weight loss were recorded during chemoradiation. Acute and long-term toxicities were graded according to Radiotherapy Oncology Group (RTOG) group severity scale (http://ctep.cancer.gov).

All patients had a follow-up visit one month and regularly three months following treatment. Clinical examination and direct endoscopic exam were performed at each follow-up to detect recurrent disease. A PET scan or PET-CT scan were performed four months, ten months and yearly after treatment if there was no evidence of disease. PET positive areas were biopsied to detect recurrence and surgery and/or chemotherapy were carried out for salvage if the biopsy demonstrated disease recurrence. Patient ability to resume normal oral feeding and dependency on tube feedings was also evaluated at each visit.

Survival data was analyzed using Kaplan-Meier estimation.

## Results

We identified 33 patients with invasive squamous cell carcinoma of the oropharynx treated at the University of Arizona Radiation Oncology department from 2007 to 2011.

Median age at diagnosis was 61 years-old (range: 39-83 years-old). There were 31 males and 2 female. There was 7 stage III, 19 stage IVA, 5 stage IVB and two stage IVC. Treatment consisted of: radiotherapy alone (1), postoperative chemoradiation (5), and definitive concurrent chemoradiation (27). The patient who had radiotherapy alone did not have chemotherapy because of significant co-morbidity with recurrent pneumonia prior to treatment. Except for two patients, all patients had a smoking history. Three patients had HPV 16 testing because of their young age and absence of smoking history. Two of these three patients were HPV 16 positive. Radiotherapy technique consisted of WF IGRT on the helical Tomotherapy unit. Table 1 summarizes patient characteristics. Table 2 summarizes radiation dose distributions among various OAR and to PTV1-3.

**Table 1:** Patient characteristics.

Patient Number		33
Age	Median	61
	Range	39–84
Sex	Male	31
	Female	2
Histology	Squamous	33
Tumor Sites	Tonsils	19
	Base of Tongue	13
	Soft palate	1
Stages	III	7
	IVA	19
	IVB	5
	IVC	2
T stages	T1	5
	T2	11
	T3	10
	T4	7
Neck nodes	N0	2
	N1	11
	N2	17
	N3	3
Treatment	Radiotherapy alone	1
	Postoperative chemoradiation	5
	Chemoradiation	27
Smoking history	>50-pack year history	31
Follow-up (months)	Median	28
	Range	6–43

**Table 2:** Dose distribution to target volume and to critical organs at risk for complications following image-guided radiotherapy for head and neck cancer.

PTV1	Mean	97.1%
	Range	95–99%
PTV2	Mean	96.9%
	Range	95–99%
PTV3	Mean	96.3%
	Range	95–99%
Spinal cord	Maximum	36.7 Gy
	Range	28.9–42 Gy
Brain stem	Maximum	44.5 Gy
	Range	35.7–50.2 Gy
Right parotid	Mean	39 Gy
	Range	18.4–65.4 Gy
Left parotid	Mean	37.6 Gy
	Range	19.6–63.8 Gy
Larynx	Mean	24.5 Gy
	Range	17.3–45.5 Gy
Right cochlea	Mean	6.9 Gy
	Range	3.6–14.3 Gy
Left cochlea	Mean	8.7 Gy
	Range	4–28.4 Gy
Mandible	Maximum	70.4 Gy
	Range	64.8–74.6 Gy
	Mean	47.3 Gy
	Range	38.8–60 Gy

PTV1: target volume receiving 66 to 70 Gy; PTV2: target volume receiving 59.6 to 63 Gy; PTV3: target volume receiving 54 to 56 Gy; Gy: gray.

Chemotherapy consisted of cisplatin (P) 30 mg/m2 intravenously (IV) weekly (21) and cisplatin 100 mg IV on day 1, 22, and 43 of radiotherapy (7). One patient had carboplatin IV weekly with an area under the curve (AUC) of 1.5 because poor kidney function. Three patient had induction chemotherapy with taxotere (T) 75 mg/m2 IV, cisplatin 100 mg/m2 IV followed by 5-fluorouracil (F) 1000 mg/m2 for four days, repeated every three weeks for three cycles followed four weeks later by carboplatin IV weekly with an area under the curve of 1.5 during radiotherapy. One patient had cetuximab IV 400 mg/m2 followed by 250 mg/m 2 weekly during radiotherapy because of concern that he may not tolerate conventional chemotherapy.

At a median follow-up of 32 months (6–47 months), no patient developed loco-regional recurrence. The two stages IVc patients had lung metastases on diagnosis. They had induction chemotherapy followed by concurrent chemoradiation. The lung metastases initially responded to induction chemotherapy but recurred four to six months respectively after chemoradiation requiring adjuvant chemotherapy and stereotactic body radiotherapy. One patient developed hematuria after chemoradiation. He was diagnosed to have locally advanced bladder cancer and died from the bladder cancer. The 3-year survival is estimated to be 92% for the whole group.

Twenty-four patients (72%) developed grade 3-4 toxicity mucositis. Six patients (25%) had grade 3–4 nausea and vomiting requiring IV fluid for hydration.

The patient who had recurrent pneumonia prior to radiotherapy continued to have pneumonia during treatment and required multiple hospital admissions.

Two patients did not complete radiotherapy. One patient decided against medical advice to discontinue treatment at 48 Gy because of grade 4 mucositis. One patient was involved in a car accident and treatment was stopped at 68 Gy. Eighteen patients (54%) had treatment breaks ranging from three to 28 days (median: 3 days) because of grade 3–4 toxicity. Median weight loss was 16 pounds (0–40 pounds).

Chemotherapy was not administered according to the protocol in six patients (18%).

One patient did not receive chemotherapy because of recurrent pneumonia prior to treatment and the concern for chemotherapy-induced immunosuppression. The patient who had induction TPF had 25% TPF dose reduction after the first cycle of chemotherapy because of neutropenia. Among patients who had weekly cisplatin 30 mg/m2, one patient developed acute tubular necrosis after the first week of chemotherapy which required its replacement with carboplatin, another patient had cisplatin on hold during week 5 because of severe vomiting. The patient who had weekly carboplatin did not receive carboplatin after week 5 because of severe weight loss and PEG tube malfunction.

For the whole group of 33 patients, two (6%) had prolonged tube feedings at 21 months and 29 months respectively because of severe dysphagia and dysgueusia. They were able to discontinue tube feedings afterward. One patient (3%) had soft tissue mucosal ulceration which resolved with antibiotics and oral hygiene.

## Discussion

To our knowledge, this is the first study looking at the feasibility of chemoradiation for locally advanced oropharyngeal cancer with tomotherapy-based IGRT and PET imaging. Even though the patient number is small with a relatively short follow-up, all patients achieved clinical and PET proven loco-regional control. We include two patients who had lung metastases at diagnosis because they were in remission following induction chemotherapy and required radiotherapy for local control. Even though the lung metastases recurred after chemoradiation, they did not have loco-regional failure even though they both had large size primaries and neck nodes (T4,N3). Even though we lacked information on HPV 16 status in most patients, the majority of the patients had a history of heavy smoking which conferred a poor prognosis. Tomotherapy is able to deliver a high radiation dose with rapid fall off. In addition, pre-treatment megavolt (MV) CT performed before treatment and re-positioning to compensate for body motion and tumor shrinkage decreased the risk of marginal miss [13]–[17].

**Figure 1:**
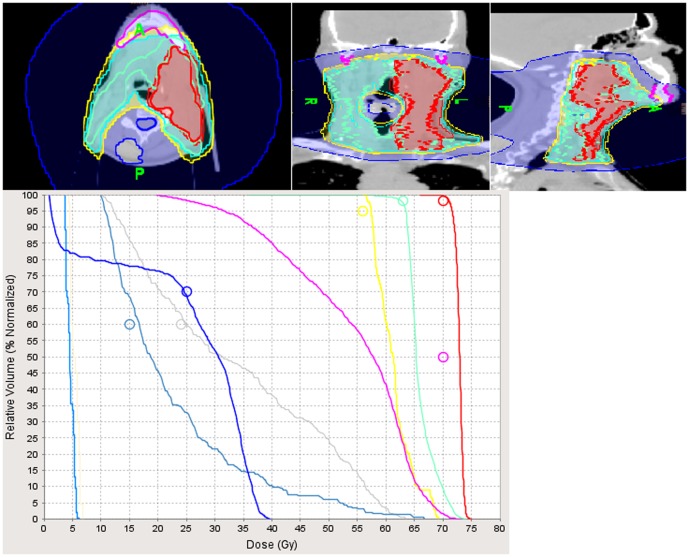
Illustration of the effectiveness of Tomotherapy to deliver high radiation dose to the gross tumor and cervical lymph nodes while sparing adjacent normal structures. The patient had locally advanced base of the tongue cancer (T4) associated with massive cervical metastases (N3) and lung metastases at diagnosis. Following induction chemotherapy which resulted in resolution of the lung metastases, he had concurrent chemoradiation for local control and achieved a complete response of the gross tumor and lymph nodes on post-treatment PET-CT. The lung metastases recurred after treatment and were treated with adjuvant chemotherapy and consolidation stereotactic body radiotherapy. The patient is currently on remission two years after the treatment with no long-term complications except for xerostomia because of low radiation dose to the normal organs. The parotid glands could not be spared because of the close proximity to the gross lymph nodes and areas at high risk for disease.Red line: gross tumor and cervical lymph nodes treated to 70 Gy; green line: area at high risk for disease treated to 63 Gy; pink line: mandibular dose (mean: 56 Gy), gray line: pharyngeal muscles dose (mean: 33.6 Gy); gray-blue line: laryngeal dose (mean: 22.5 Gy); navy blue line: spinal cord dose (max: 39.4 Gy); light blue line: right cochlea dose: (mean: 4.5 Gy); light brown line: left cochlea dose: (mean: 5.3 Gy).

As we reported previously, the study mean laryngeal and cochlea dose remained consistently low [16], [18]. Low dose to the larynx may reduce the risk of laryngeal edema and preserve voice quality. Decreased cochlea dose may decrease the risk of hearing loss and potentially improve patient quality of life because of the proven ototoxicity of cisplatin-based chemotherapy. The mean parotid dose was high because most of the patients presented with cervical lymph node metastases at diagnosis. It is our policy not to spare the ipsilateral parotid gland in case of neck node involvement because of the risk recurrence in the peri-parotid area [19]. As a result, loco-regional control was excellent despite locally advanced disease. For instance, Figure 1 illustrated a patient with a T4N3M1 (IVc) base of tongue carcinoma who presented with lung metastases at diagnosis. Following induction chemotherapy with TPF, the patient underwent concurrent chemoradiation with complete resolution of the gross tumor and cervical lymph nodes on follow-up PET-CT. The lung metastases were treated with adjuvant chemotherapy and stereotactic body irradiation. The patient is currently on remission two years after treatment with no long-term complications because of the low dose of radiation to the normal tissues. Even though the study follow-up is relatively short, PET-CT following treatment allows us to detect disease recurrence or second primaries effectively [20]–[23]. Indeed, the patient who had biopsy-proven bladder cancer was diagnosed on PET-CT. PET-CT may also have predictive prognostic value. In a study of 80 head and neck cancer patients who underwent radiotherapy and were monitored with PET-CT, the patients who had a negative PET-CT within six months of treatment completion had a 2-year disease-free survival of 93% [20]. The high accuracy rate of PET-CT in predicting survival benefit for patients who were disease-free by PET criterias following radiotherapy or chemoradiation for head and neck cancer was also corroborated in other studies [21]–[23]. Thus, we feel confident that the excellent loco-regional control observed in our study may be maintained with a longer follow-up.

We observe a high rate of grade 3–4 mucositis and gastrointestinal toxicity during treatment. The combination of chemotherapy and radiation for locally advanced oropharyngeal cancer is frequently associated with significant acute toxicity because of the large volume of normal tissues irradiated even with IMRT [11], [24]. However, the acute toxicity frequently resolved by four to six weeks after chemoradiation. We have a special team of dietitians, speech pathologists, and home health nurses who monitored patients closely during and after treatment because of the significant weight loss secondary to mucositis. Only two patients (6%) became dependent on long-term tube feedings because chronic dysphagia and dysgueusia. They eventually recovered andhad removal of the feedings tubes. If we look at the long-term toxicity of patients with locally advanced oropharyngeal cancer treated with concurrent chemotherapy and conventional radiotherapy, up to 37% of the patients had prolonged tube feedings after treatment because of severe dysphagia or aspiration [9]. Long-term dependence on feeding tubes was also observed in 14% of the patients treated with IMRT and chemotherapy for oropharyngeal cancer [11]. Even with a special IMRT technique designed to reduce irradiation to the larynx and pharyngeal muscles which are critical structures for swallow, four out of 76 patients (5%) still experienced significant dysphagia one year following chemoradiation for oropharyngeal cancer [25].

Thus, our treatment complications profile compares favorably with other studies on oropharyngeal cancers and may improve further in the future as we acquire more experience with this new technique of radiotherapy. We also consider the administration of amifostine, a radiation protector, during chemoradiation for oropharyngeal cancers in the future to reduce the severity of mucositis and long-term dysphagia [26], [27].

The limitations of the present study include the retrospective nature of the study, the small number of patients, the lack of HPV 16 information on most patients, and the relatively short follow-up. We do not have a matched cohort of oropharyngeal cancer patients treated with the conventional radiotherapy technique because these patients did not have a dose-volume histogram for comparison. However, our study demonstrates the feasibility of tomotherapy treatment for local control in patients with locally advanced oropharyngeal cancers with acceptable toxicity. Further prospective studies with a large number of patients should be performed with tomotherapy to assess the impact of this new technique on patient quality of life because of the unique ability of Tomotherapy to decrease radiation dose to the normal tissues.

## Conclusions

Tomotherapy-based IGRT provides excellent loco-regional control and in patients with locally advanced oropharyngeal carcinoma with acceptable long-term toxicity. However, acute toxicity, mainly mucositis remains significant and should be taken into consideration in future prospective trials.

## References

[1] Shiboski CH, Schmidt BL, Jordan RC (2005) Tongue and tonsil carcinoma. Increasing trends in the U.S. population ages 20–44 years. Cancer 103: 1843–1849. 15772957 10.1002/cncr.20998

[2] Nguyen NP, Chi A, Nguyen LM, Ly BH, Karlsson U, et al (2010) Human papillomavirus-associated oropharyngeal cancer: a new clinical entity. Q J Med 103: 229–236. 10.1093/qjmed/hcp176 20015950

[3] Jemal A, Siegel R, Ward E, Hao Y, Xu J, et al (2008) Cancer statistics, 2008. CA Cancer J Clin 58: 71–96. 18287387 10.3322/CA.2007.0010

[4] Soo KC, Tan EH, Wee J, Lim D, Tai BC, et al (2005) Surgery and adjuvant radiotherapy vs concurrent chemoradiotherapy in stage III/IV nonmetastatic squamous cell carcinoma of the head and neck: a randomized comparison. Br J Cancer 93: 279–286. 16012523 10.1038/sj.bjc.6602696 PMC2361563

[5] Lauciello FR, Vergo T, Schaaf NG, Zimmerman R (1980) Prosthodontic and speech rehabilitation after partial and complete glossectomy. J Prosthet Dent 43: 204–211. 6985967 10.1016/0022-3913(80)90188-2

[6] Pauloski BR, Rademaker AW, Logemann JA, McConnell FM, Heiser MA, et al (2004) Surgical variables affecting swallowing in patients treated for oral/oropharyngeal cancer. Head Neck 26: 625–636. 15229906 10.1002/hed.20013

[7] Kuntz AL, Weymuller EA (1999) Impact of neck dissection on quality of life. Laryngoscope 8: 1334–1338. 10.1097/00005537-199908000-00030 10443845

[8] Parsons JT, Mendenhall WM, Stringer SP, Amdur RJ, Hinerman RW, et al (2002) Squamous cell carcinoma of the oropharynx: surgery, radiation therapy, or both. Cancer 94: 2967–2980. 12115386 10.1002/cncr.10567

[9] Nguyen NP, Vos P, Smith HJ, Ly BH, Karlsson U, et al (2007) Concurrent chemoradiation for locally advanced oropharyngeal cancer. Am J Otolaryngol 28: 3–8. 17162122 10.1016/j.amjoto.2006.03.007

[10] Nguyen NP, Frank C, Moltz CC, Vos P, Smith HJ, et al (2009) Analysis of factors influencing aspiration risk following chemoradiation for oropharyngeal cancer. Br. J Radiol 82: 675–680. 10.1259/bjr/72852974 19332514

[11] de Arruda FF, Puri DR, Zhung J, Narayana A, Wolden S, et al (2006) Intensity-modulated radiation therapy for the treatment of oropharyngeal carcinoma: The Memorial Sloan Kettering center experience. Int J Radiat Oncol Biol Phys 64: 363–373. 15925451 10.1016/j.ijrobp.2005.03.006

[12] Lee TF, Fang FM, Chao PJ, Su TJ, Wang LK, et al (2008) Dosimetric comparisons of helical tomotherapy and step-and-shoot intensity-modulated radiotherapy in nasopharyngeal carcinoma. Radiother Oncol 89: 89–96. 18524401 10.1016/j.radonc.2008.05.010

[13] Jacob V, Bayer W, Astner ST, Kneschaurek P (2010) A planning comparison of dynamic IMRT for different collimator leaf thickness with helical tomotherapy and RapidArc for prostate and head and neck tumors. Strahlenther Onkol 186: 502–510. 20803184 10.1007/s00066-010-2124-3

[14] Murthy V, Master Z, Gupta T, Ghosh-Laskar S, Buddrukkar A, et al (2010) Helical tomotherapy for head and neck squamous cell carcinoma: Dosimetric comparison with linear accelerator-based step-and-shoot IMRT. J Cancer Res Treat 6: 194–198. 10.4103/0973-1482.65245 20622367

[15] Chen AM, Lee NY, Yang CC, Liu T, Naravan S, et al (2010) Comparison of intensity-modulated radiotherapy using helical tomotherapy and segmental multileaf collimator-based techniques for nasopharyngeal carcinoma: dosimetric analysis incorporating quality assurance guidelines from RTOG 0225. Technol Cancer Res Treat 9: 291–298. 20441239 10.1177/153303461000900308

[16] Nguyen NP, Ceizyk M, Vos P, Vinh-Hung V, Davis R, et al (2010) Effectiveness of image-guided radiotherapy for laryngeal sparing in head and neck cancer. Oral Oncol 46: 283–286. 20188620 10.1016/j.oraloncology.2010.01.010

[17] Ulrich S, Sterzing F, Nill S, Schubert K, Herfarth KK, et al (2009) Comparison of arc-modulated cone beam therapy and helical tomotherapy for three different types of cancer. Med Phys 36: 4702–4710. 19928101 10.1118/1.3223633

[18] Nguyen NP, Smith-Raymond L, Vinh-Hung V, Sloan D, Davis R, et al (2011) Feasibility of Tomotherapy to save the cochlea from excessive radiation in head and neck cancer. Oral Oncol 47: 414–419. 21474364 10.1016/j.oraloncology.2011.03.011

[19] Cannon DM, Lee NY (2008) Recurrence in region of spared parotid gland afterdefinitive intensity-modulated radiotherapy for head and neck cancer. Int J Radiat Oncol Biol Phys 70: 660–665. 18037580 10.1016/j.ijrobp.2007.09.018

[20] Salaun PY, Abgral R, Querellou S, Couturier O, Valette G, et al (2007) Does 18-fluorodeoxyglucose positron emission tomography improve recurrence detection in patients treated for head and neck squamous cell carcinoma with negative clinical follow-up? Head Neck 29: 1125–1130. 10.1002/hed.20645 17636537

[21] Malone JP, Gerberi MAT, Vasireddy S, Hughes LF, Rao K, et al (2009) Early prediction of response to chemoradiotherapy for head and neck cancer. Arch Otolaryngol Head Neck Surg 135: 1119–1125. 19917925 10.1001/archoto.2009.152

[22] Yao M, Smith RB, Graham MM, Hoffman HT, Tan H, et al (2005) The role of FDG PET in management of neck metastases from head and neck cancer after definitive radiotherapy treatment. Int J Radiat Oncol Biol Phys 63: 991–999. 16099601 10.1016/j.ijrobp.2005.03.066

[23] Ong SC, Schoder H, Lee NY, Patel SG, Carlson D, et al (2008) Clinical utility of 18F-FDG PET/CT in assessing the neck after concurrent chemoradiotherapy for locoregionally advanced head and neck cancer. J Nucl Med 49: 532–540. 18344440 10.2967/jnumed.107.044792

[24] Daly ME, Le QT, Maxim PG, Loo BW, Kaplan MJ, et al (2010) Intensity-modulated radiotherapy in the treatment of oropharyngeal cancer: clinical outcomes and pattern of failure. Int J Radiat Oncol Biol Phys 76: 1339–1346. 19540068 10.1016/j.ijrobp.2009.04.006

[25] Feng FY, Kim HM, Lyden TH, Haxer MJ, Worden FP, et al (2010) Intensity-modulated chemoradiotherapy aiming to reduce dysphagia in patients with oropharyngeal cancer: clinical and functional results. J Clin Oncol 28: 2732–2738. 20421546 10.1200/JCO.2009.24.6199 PMC2881852

[26] Buntzel J, Glatzel M, Mucke R, Micke O, Bruns F (2007) Influence of amifostine on late radiation-toxicity in head and neck cancer—a follow-up study. Anticancer Res 27: 1953–1956. 17649803

[27] Buntzel J, Kuttner K, Frolich D, Glatzel M (1998) Selective cytoprotection withamifostine in concurrent radiochemotherapy for head and neck cancer. Ann Oncol 9: 505–509. 9653491 10.1023/a:1008282412670

